# Supplementary material for: CSN5A Subunit of COP9 Signalosome Temporally Buffers Response to Heat in Arabidopsis
Source: Biomolecules. 2019 Nov 29;9(12):805. doi: 10.3390/biom9120805 (PMC6995552; doi:10.3390/biom9120805)
Supplement: Supplementary file 1 [file biomolecules-09-00805-s001.pdf]

The following Supporting Information is available for this article:

Figure S1. Phenotypic study showing time series of rosette area (mm^2) of photo-morphogenesis mutants after 7 d heat stress (2h, 44ºC, 14 - 21 DAS).

Figure S2. Number of lateral roots increases in *csn5a-1* seedlings following 7 d heat treatment.

Figure S3. Change in the percentage of leaf greenness in *csn5a-1* mutant after heat stress is different from Col-0.

Figure S4. Image of mesophyll cells and size in 6th true leaf of 30 DAS seedlings undergone 7 d heat treatment.

Figure S5. Heat map showing the distribution of differentially up-regulated pathways.

Figure S6. Network association of genes upregulated in *csn5a-1* heat compared to WT heat.

Table S1. Primers used in this research work.

Table S2. Fold change of deneddylated vs neddylated cullin following heat treatment.

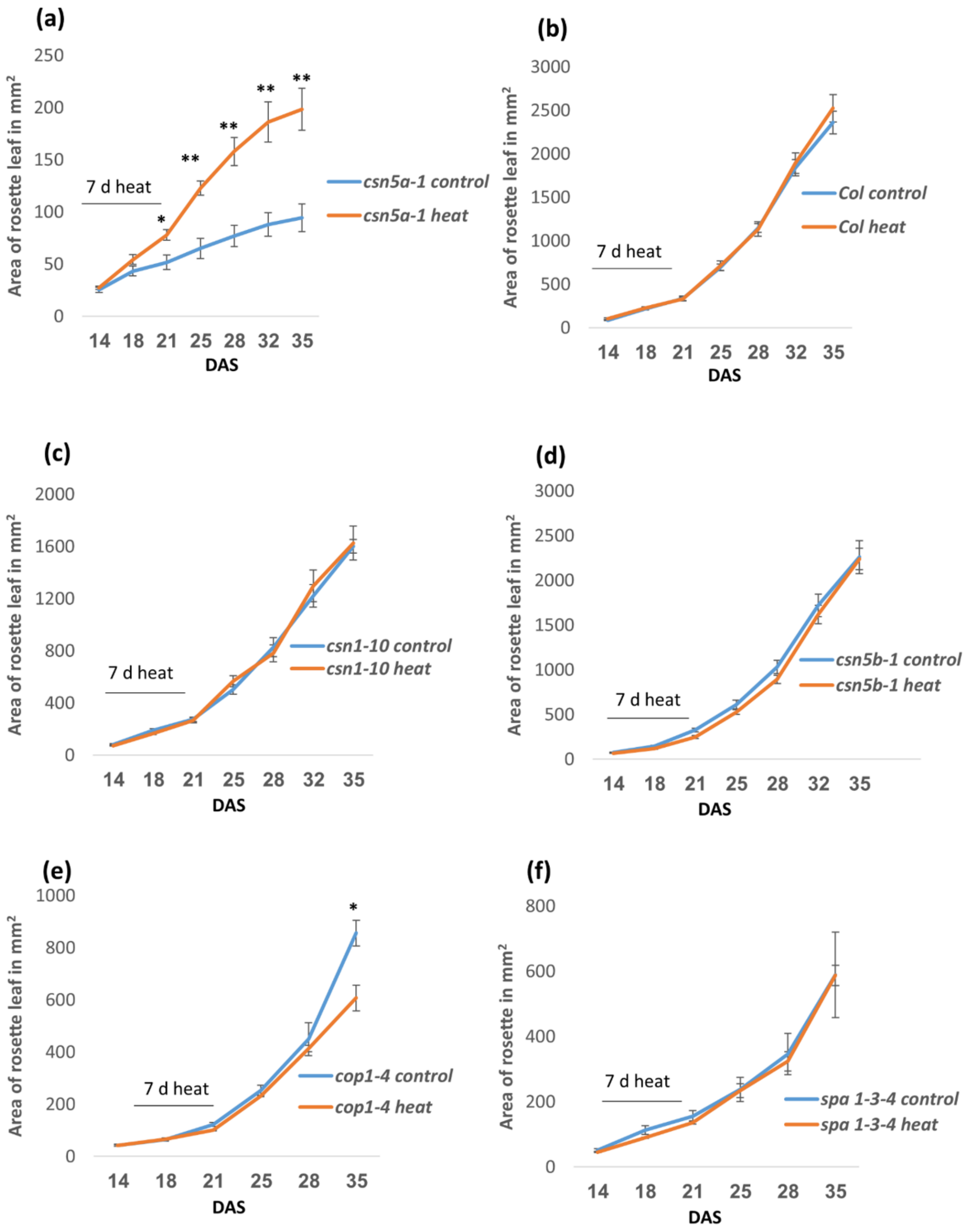

Figure S1. Phenotypic study showing time series of rosette area (mm^2) of photo-morphogenesis mutants after 7 d heat stress (2h, 44ºC, 14 - 21 DAS). (**a**) *csn5a-1* (**b**) Col-0, (**c**) *csn1-10*, (**d**) *csn5ba-1*, (**e**) *cop1-4*, and (**f**) *spa1-3-4* after 7 d heat stress compared to unstressed. Error bars represent SEM of biological replicates (n=4). Student's t test *, $P < 0.05$; **, $P < 0.01$

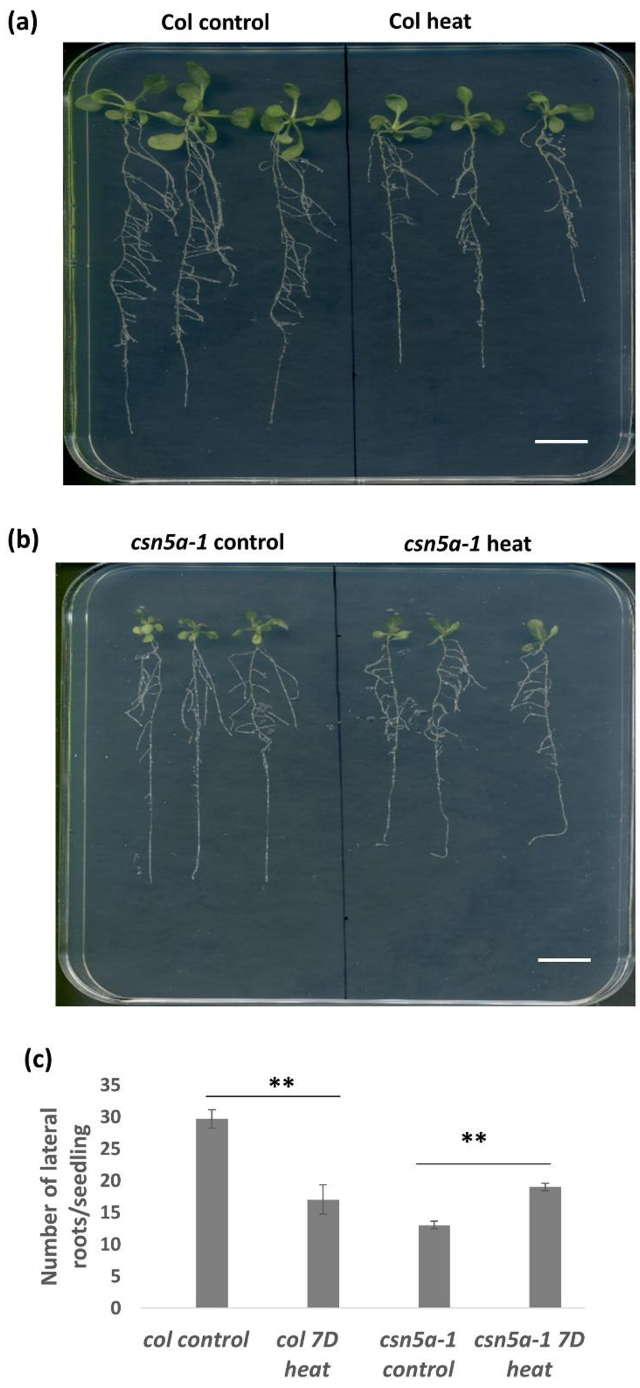

Figure S2. Number of lateral roots increases in *csn5a-1* seedlings following 7 d heat treatment. (a) Number of lateral root decreases in Col-0 after heat treatment. (b) Number of later roots increase in *csn5a-1* following heat treatment. (c) Graph displaying a change in the number of later roots in Col-0 and *csn5a-1* after heat treatment. Error bars represent SEM of biological replicates (n=3). Student's t test **, $P < 0.01$. Bars, 1cm.

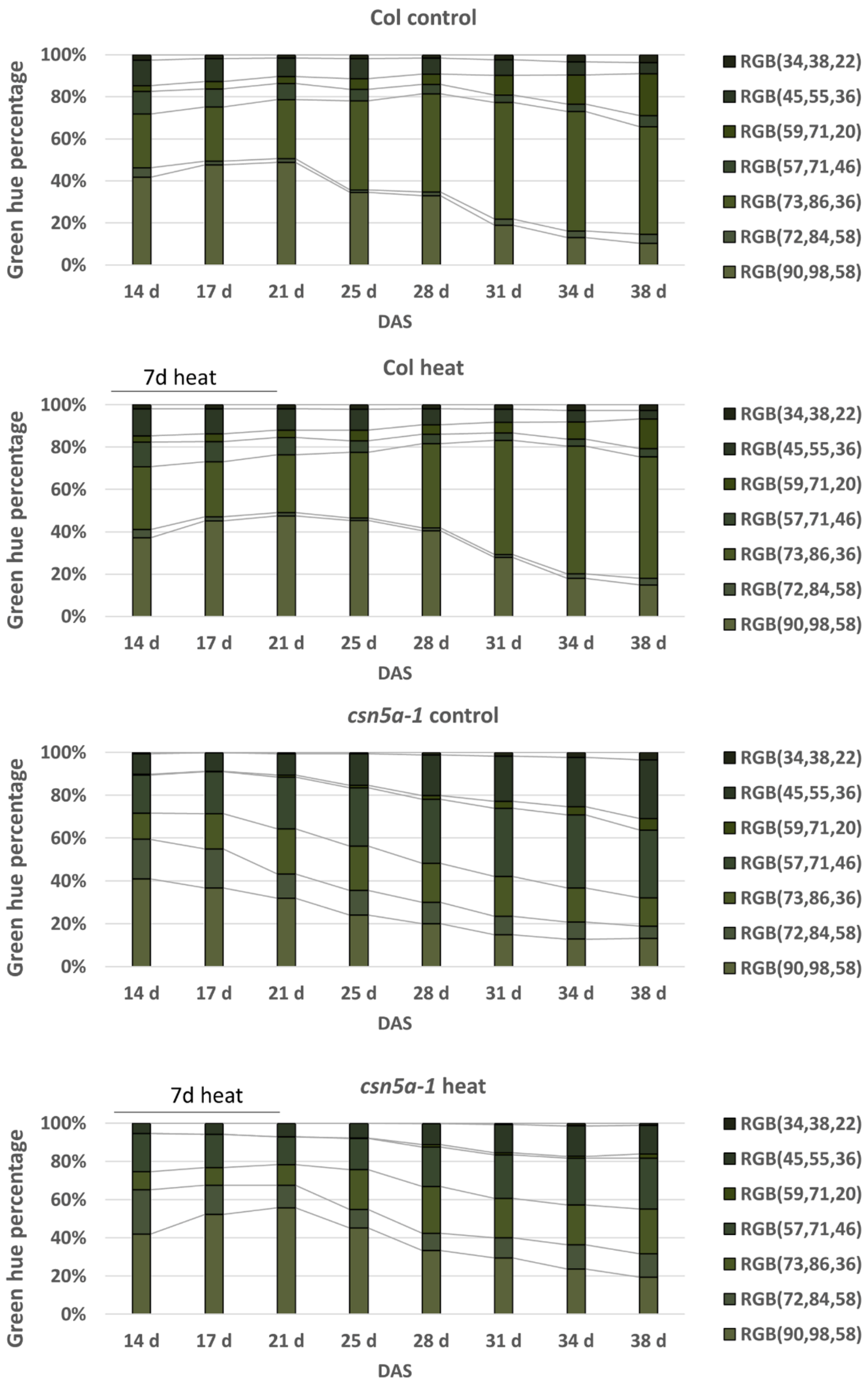

Figure S3. Change in the percentage of leaf greenness in *csn5a-1* mutant after heat stress is different from Col-0.

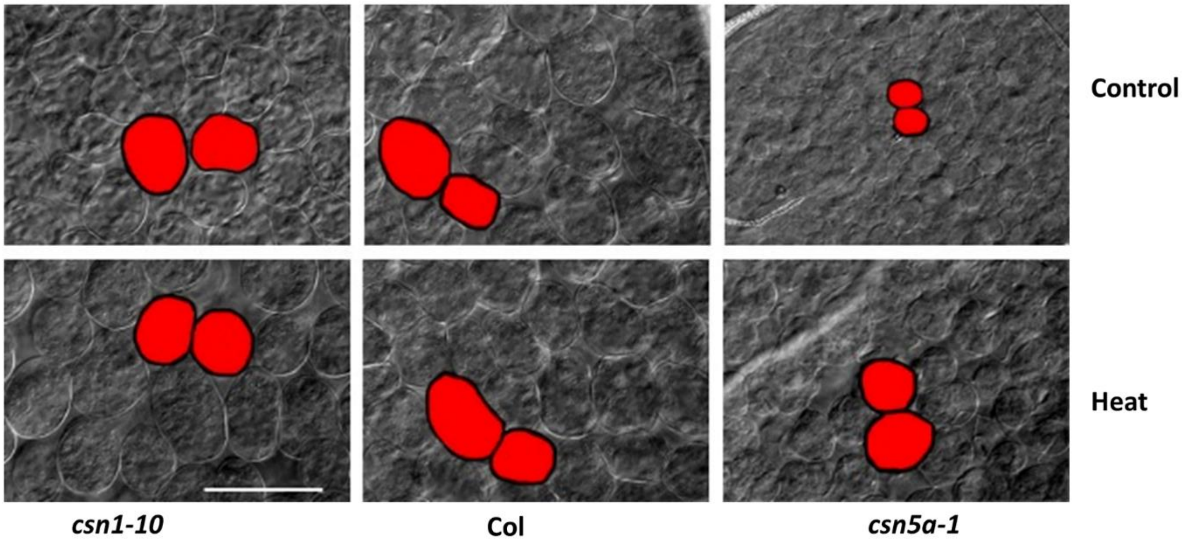

Figure S4. Image of mesophyll cells and size in 6th true leaf of 30 DAS seedlings undergone 7 d heat treatment. Mesophyll cell size increases in *csn5a-1* following heat stress but not in Col-0 and *csn1-10*. Red color is used to represent the cell area.

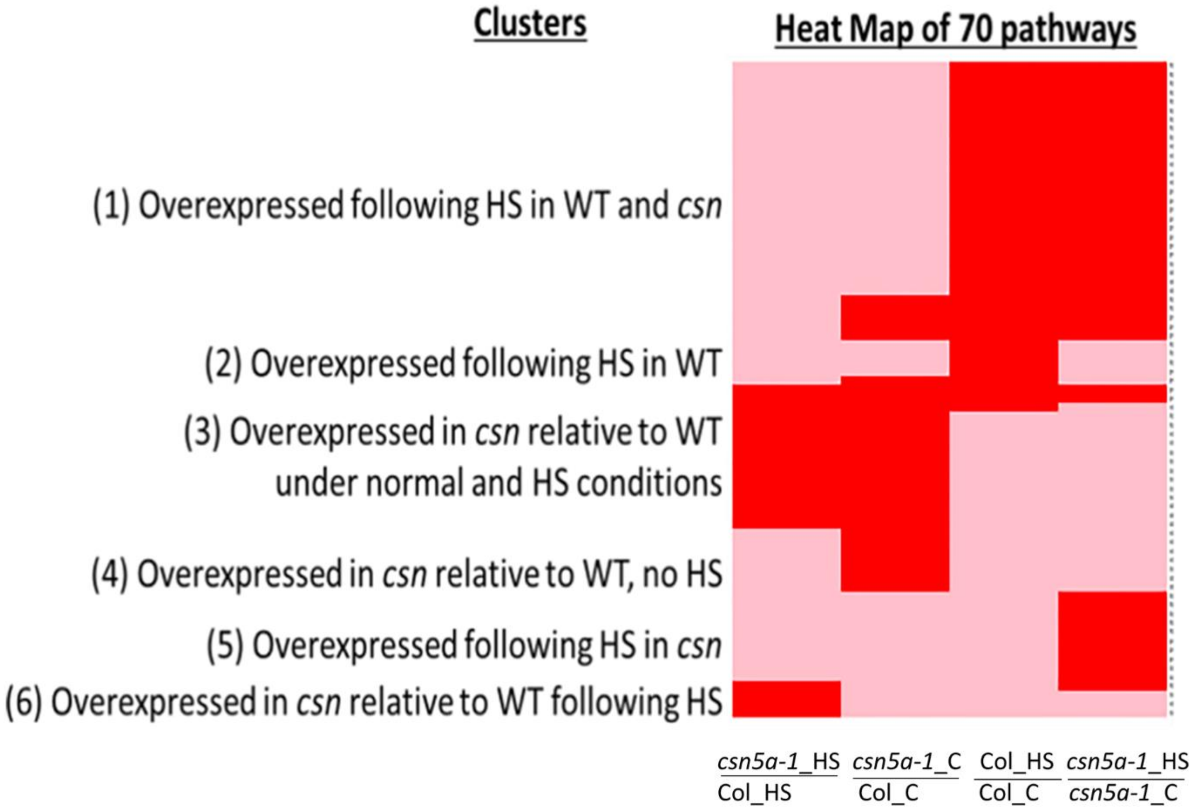

Cluster	Enriched pathways
4	Stress; ABA; Brassinosteroids
5	Histone acetyl-transferases; Transcription factors; E3 ligases; Sugar signaling Light signaling
6	Signaling receptor kinases; Auxin

Figure S5. Heat map showing the distribution of differentially up-regulated pathways. Expression pattern from *csn5a-1* mutant compared to WT following heat stress (38ºC, 3 h, 1 d); RNA sequencing was performed from four different samples 1. Untreated WT 2. Heat stressed (HS) WT 3. Untreated *csn5a-1* 4. HS *csn5a-1*. Transcriptome analysis shows the overexpression of auxin in HS *csn5a-1* mutant compared to HS WT.

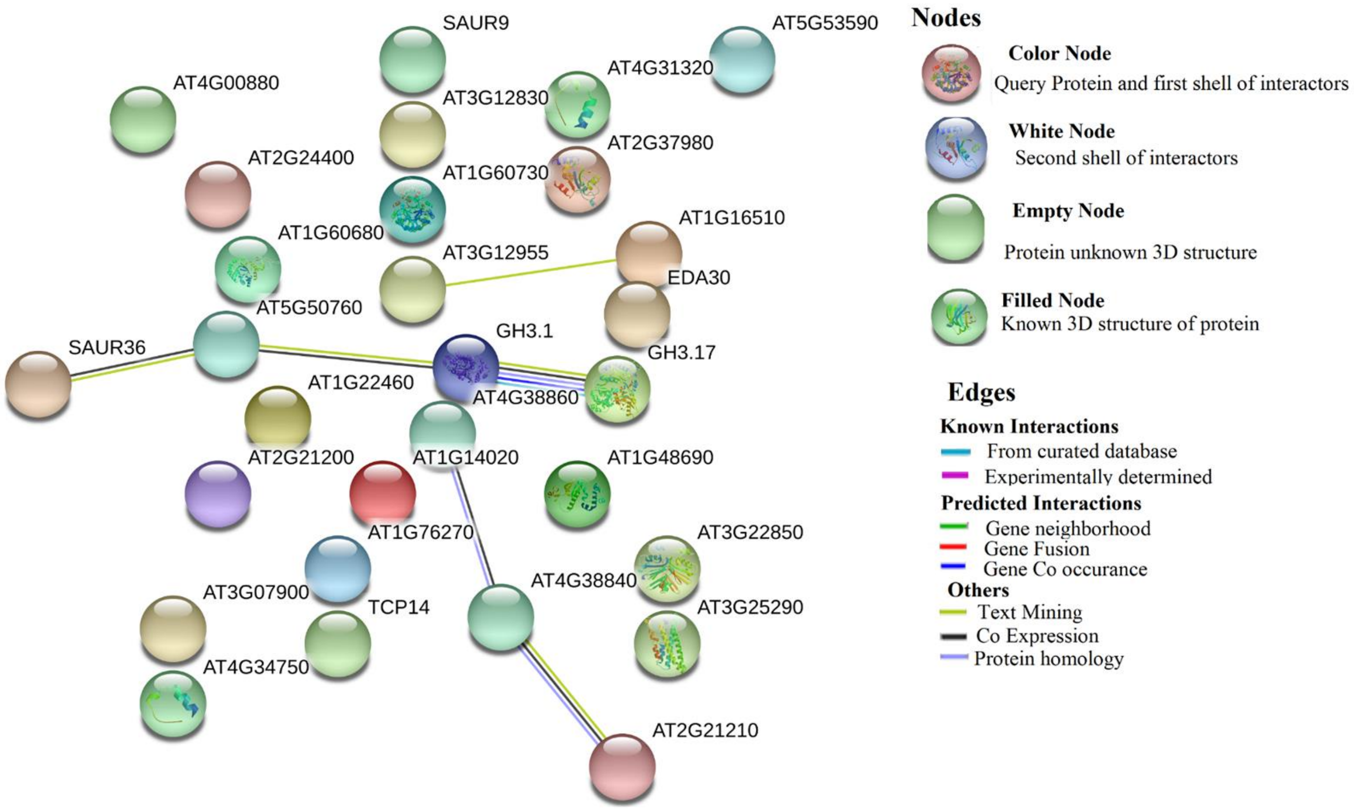

Figure S6. Network association of genes upregulated in *csn5a-1* heat compared to WT heat.

Table S1. Primers used in this research work

Gene	Primer sequence 5'- 3'
SAUR19 (AT5G18010)	F- GAAGATTCTAAGCCGCTCCA<br>R- CGGTTGGCTCAAGTATGAGAG
SAUR7 (AT2G21200)	F- AGCAAAGCAGCTTCAACACC<br>R- AGAAGGCTGGTTCAAGAACG
EXPA10 (AT1G26770)	F- CATATCTCTCTTTCTTTCTCTTGAAGG<br>R- ACCCAAGATGACCCATGTTT
EXPA4 (AT2G39700)	F- ACGCACACGCCACTTTTTAC<br>R- AAACAGCGCCGTACTCAAAG
ACTIN 8 (AT1G49240)	F- CACTTTCCAGCAGATGTGGATC<br>R- AATGCCTGGACCTGCTTCAT

Table S2. Fold change of deneddylated vs neddylated cullin following heat treatment

Samples	Neddylated	Deneddylated	CUL1/CUL1^{Nedd}	Increase in dennedylation after heat	Fold change in CUL1/CUL1^{Nedd}
**WT**	633.062	4340.388	6.856		
**WT heat**	1175.933	9641.773	8.199	1.1958	0.19
csn5a-1	3829.338	81.678	0.0213		
***csn5a-1* heat**	5614.338	726.548	0.129	6.056	5.05
csn1-10	2186.933	1526.811	0.698		
***csn1-10* heat**	2015.225	1379.74	0.685	0.98	0
